# Supplementary material for: Structures of the eukaryotic ribosome and its translational states in situ
Source: Nat Commun. 2022 Dec 2;13:7435. doi: 10.1038/s41467-022-34997-w (PMC9718845; doi:10.1038/s41467-022-34997-w)
Supplement: Supplementary file 1 — Supplementary Information [file 41467_2022_34997_MOESM1_ESM.pdf]

Supplementary Information

Structures of the eukaryotic ribosome and its translational states *in situ*

Authors: Patrick C. Hoffmann[†], Jan Philipp Kreysing[†], Iskander Khusainov[†], Maarten W. Tuijtel, Sonja Welsch and Martin Beck[*]

[†] These authors contributed equally.

* Correspondence to: martin.beck@biophys.mpg.de

Supplementary Figures

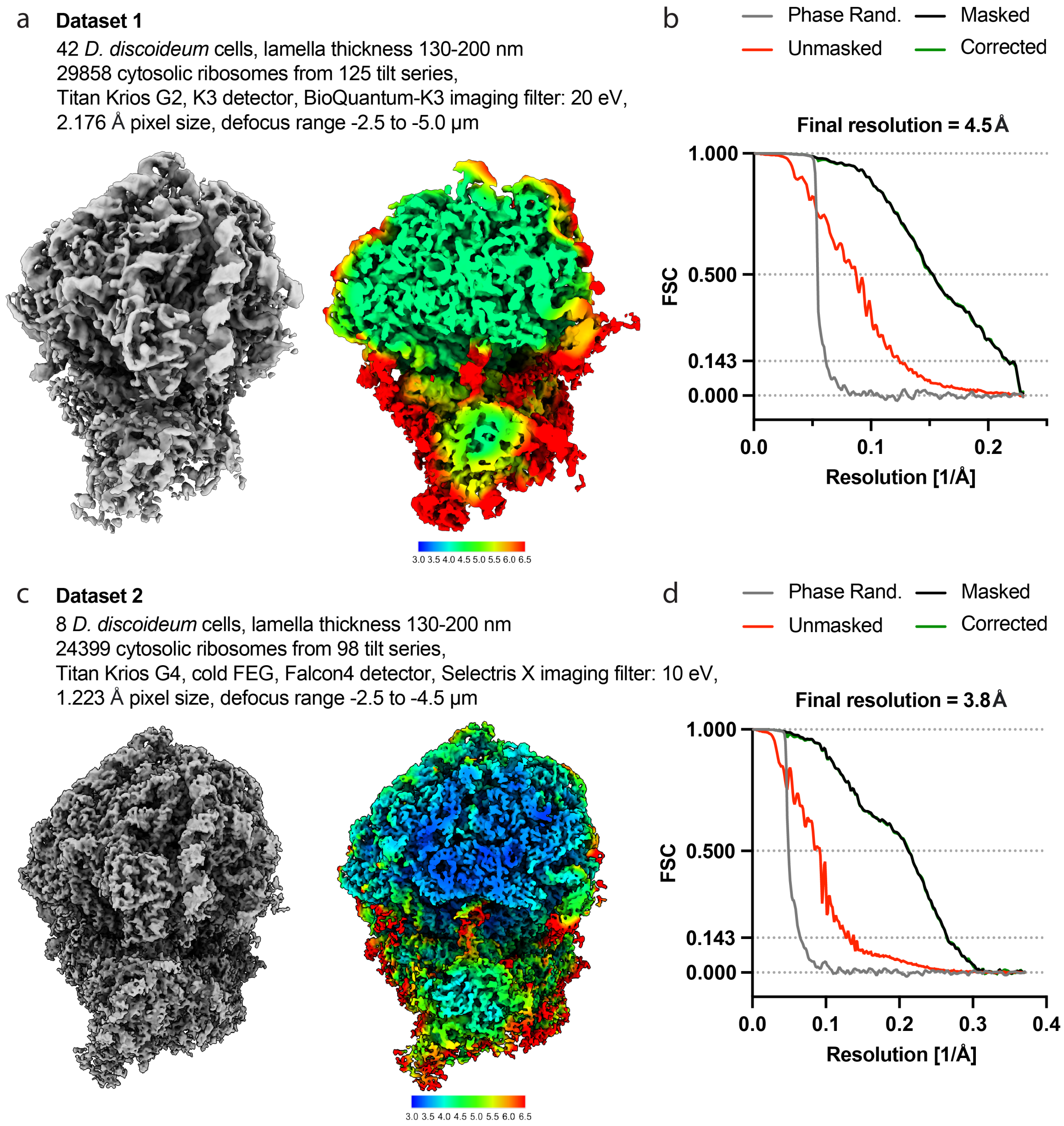

Supplementary Fig. 1. *In situ* ribosome structure of *D. discoideum*. (a) Subtomogram average of the 80S *D. discoideum* ribosome (left) obtained from dataset 1 and corresponding local resolution map (right). **(b)** Fourier shell correlation of ribosome average from dataset 1 with final resolution of 4.5 Å (29858 particles) at 0.143 cutoff. **(c)** Subtomogram average of the 80S *D. discoideum* ribosome (left) obtained from dataset 2 and corresponding local

resolution map (right). **(d)** Fourier shell correlation of ribosome average from dataset 2 with final resolution of 3.8 Å (24399 particles) at 0.143 cutoff.

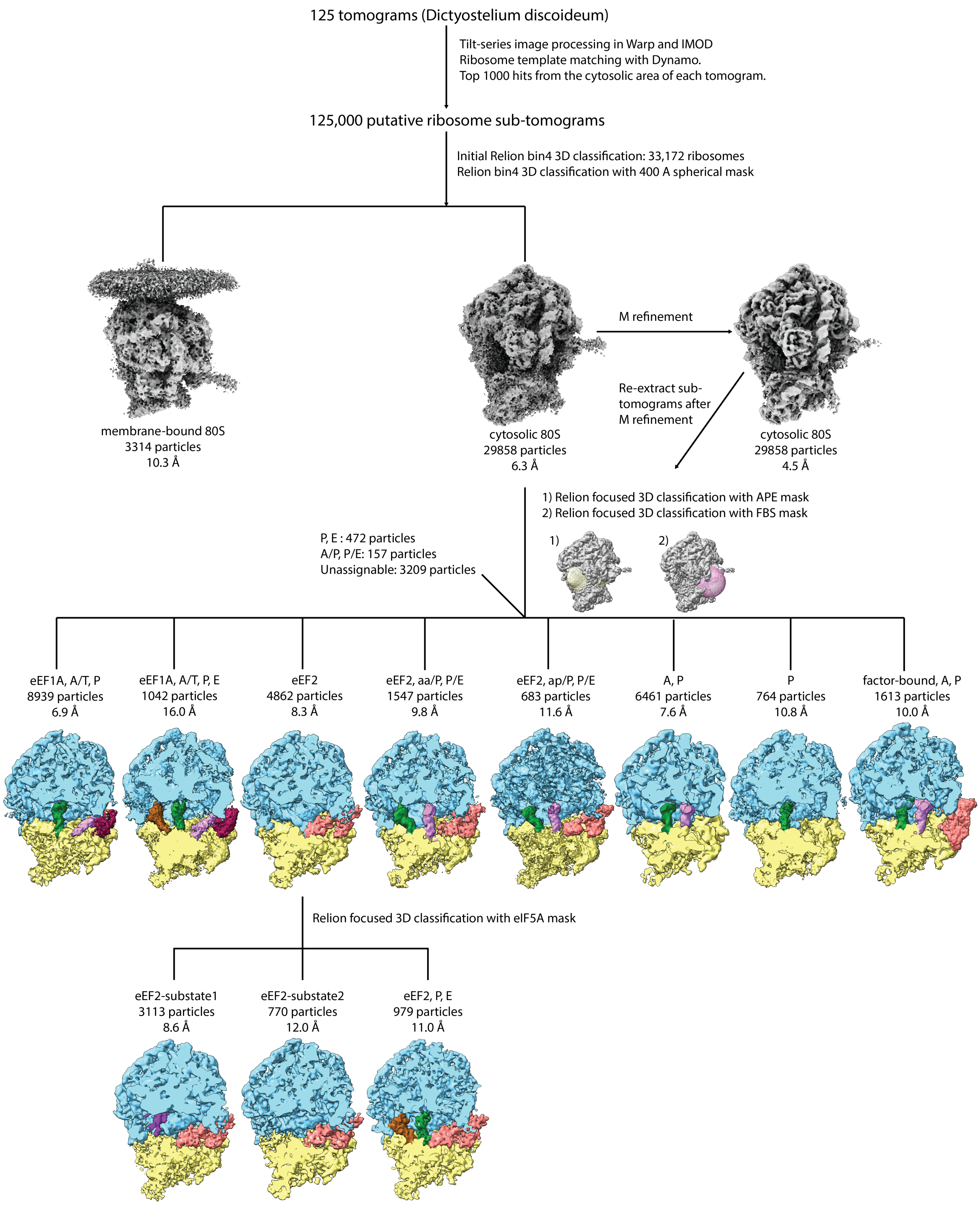

Supplementary Fig. 2. Subtomogram averaging and classification workflow for dataset 1. The classification procedure was divided into three rounds. First, global classification, second focused classification on the A-, P-, E-site tRNA region and third, focused classification on the factor binding site region. For the 80S*eEF2 state another round of focused classification

on the binding region of eIF5A was performed. The resulting states are shown with segmented maps. Color legend: Blue, 60S subunit. Yellow, 40S subunit. Lavender, A-tRNA. Green, P-tRNA. Brown, E-tRNA. Maroon, eEF1A. Salmon, eEF2. Purple, additional density in eIF5A binding region.

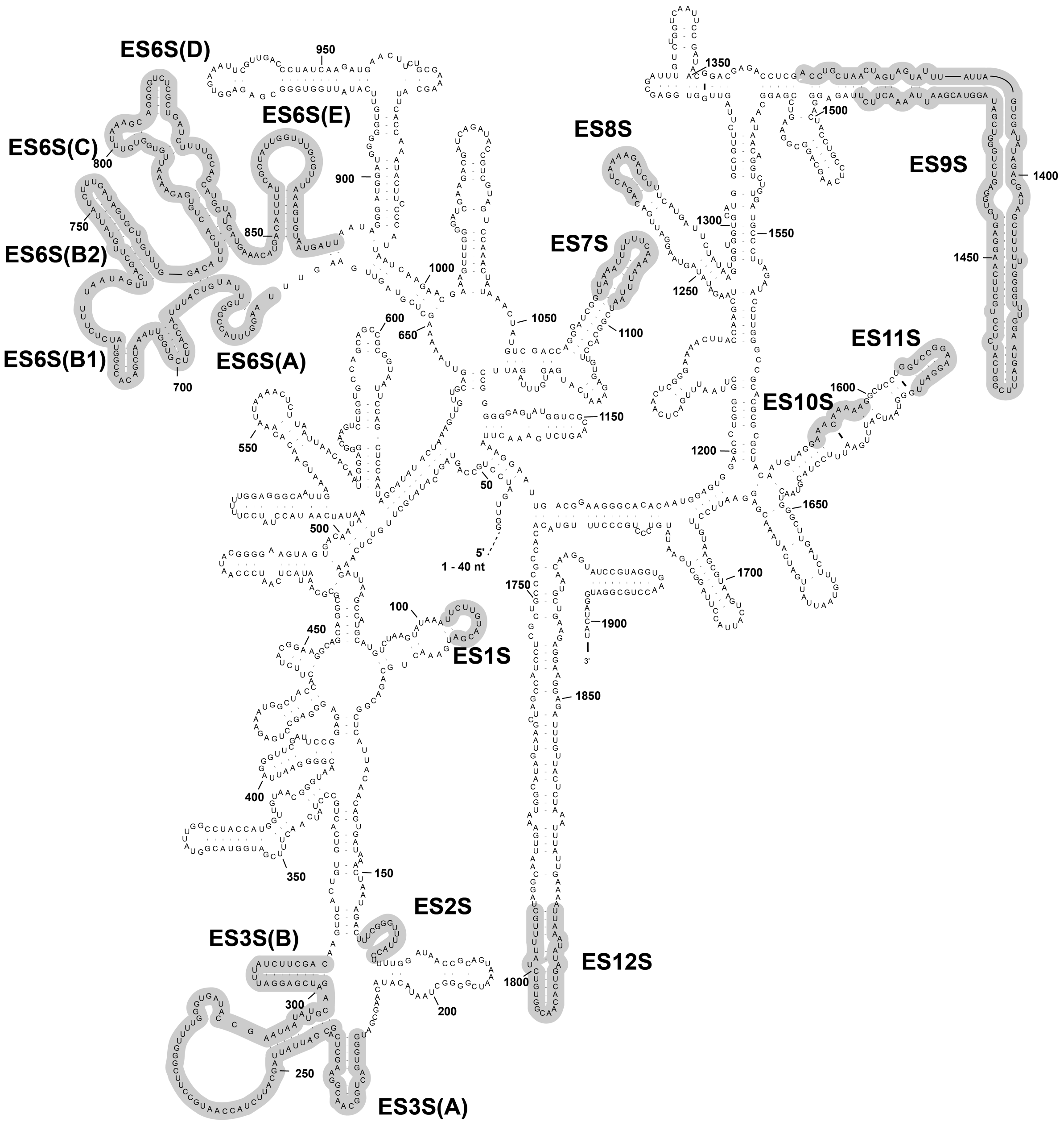

Supplementary Fig. 3. The complete 17S rRNA 2D diagram. Expansion segments are highlighted in grey. Every 50th nucleotide is labeled.

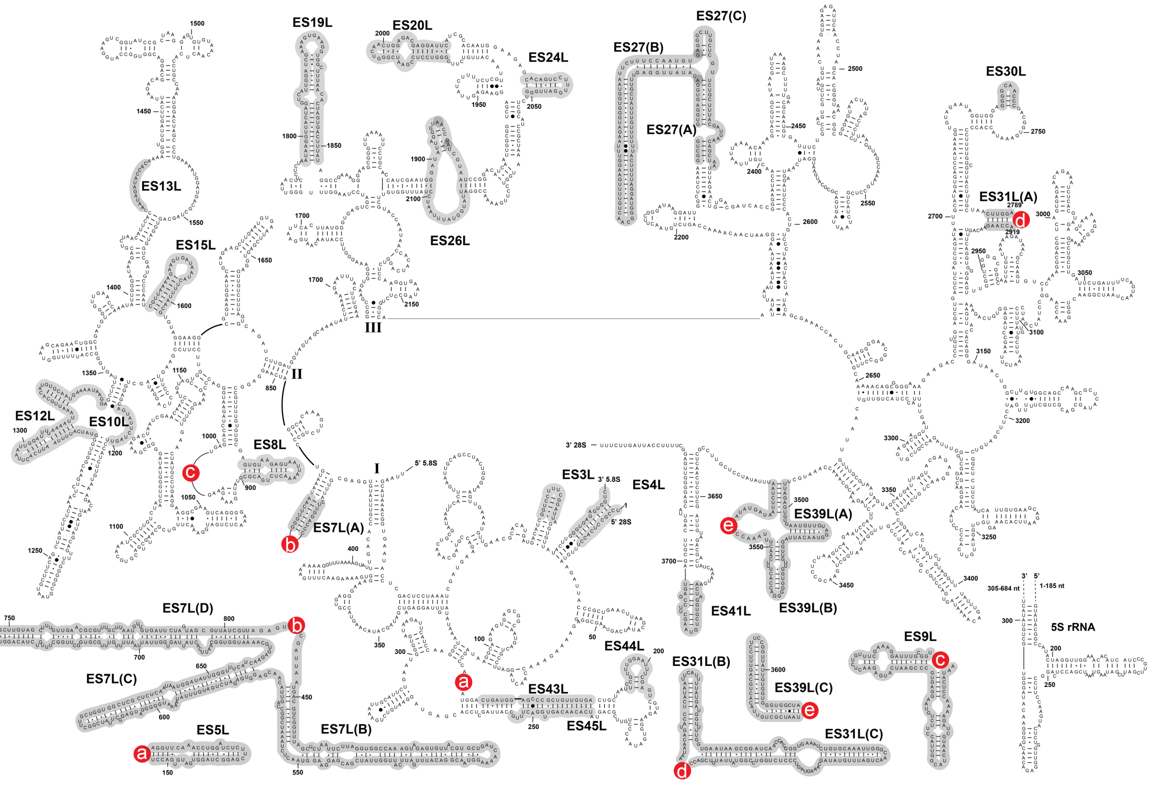

Supplementary Fig. 4. The complete 5S, 5.8S, 25S rRNA 2D diagram. Expansion segments are highlighted in grey. Every 50th nucleotide is labeled. The 80S-bound 5S rRNA does not contain residues 1-185 and 305-684, therefore they were not modeled. Parts of the continuous rRNA sequence corresponding to ES5L (a), ES7L (b), ES9L (c), ES31L(B,C) (d), ES39L (e) are highlighted in red and represented at the bottom of the diagram.

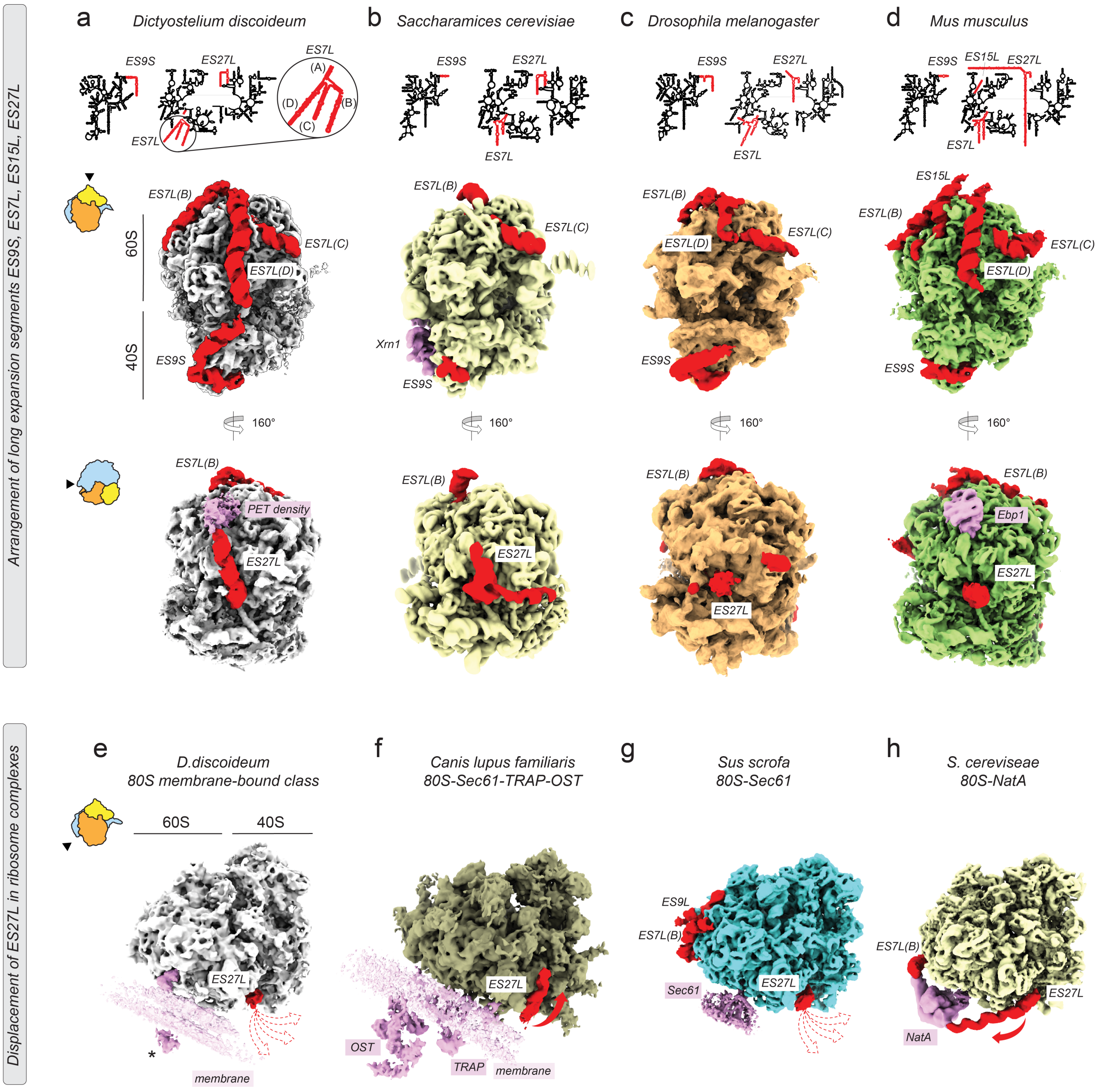

Supplementary Fig. 5. Structural comparison of the rRNA expansion segments in different model organisms. (a-d) Arrangement of the expansion segments ES9S, ES7L, ES27L on cryo-EM structures of ribosomes from *D. discoideum* (**a**, this study), **(b)** *S. cerevisiae* 80S-Xrn1 complex, EMD-4474 [7], **(c)** *D. melanogaster* 80S, EMD-5591 [8], and **(d)** *M. musculus* 80S-Ebp1 complex, EMD-10321 [9]. Upper panel represents schematic 2D diagrams of the rRNA from respective organisms. **(e-h)** Conformational changes of ES27L seen in the cryo-EM structures of ribosomes from *D. discoideum* membrane-bound class (**e**, this study), **(f)** *Canis lupus familiaris* 80S-Sec61-TRAP-OST complex bound to ER vesicles, EMD-3069 [11], **(g)** *Sus scrofa* 80S-Sec61 complex, EMD-2644 [10], and **(h)** *S. cerevisiae* 80S-

NatA complex, EMD-0202 [26]. All densities were Gaussian-filtered. Expansion segments are highlighted in red and labeled. Protein partners and ribosome-bound structures are colored in pink and labeled. The red arrows point to the direction of the ES27L protrusion. The dashed red arrows indicate the flexibility of the ES27L. Asterisk marks the presumable TRAP complex density on *D. discoideum* ribosome in panel e.

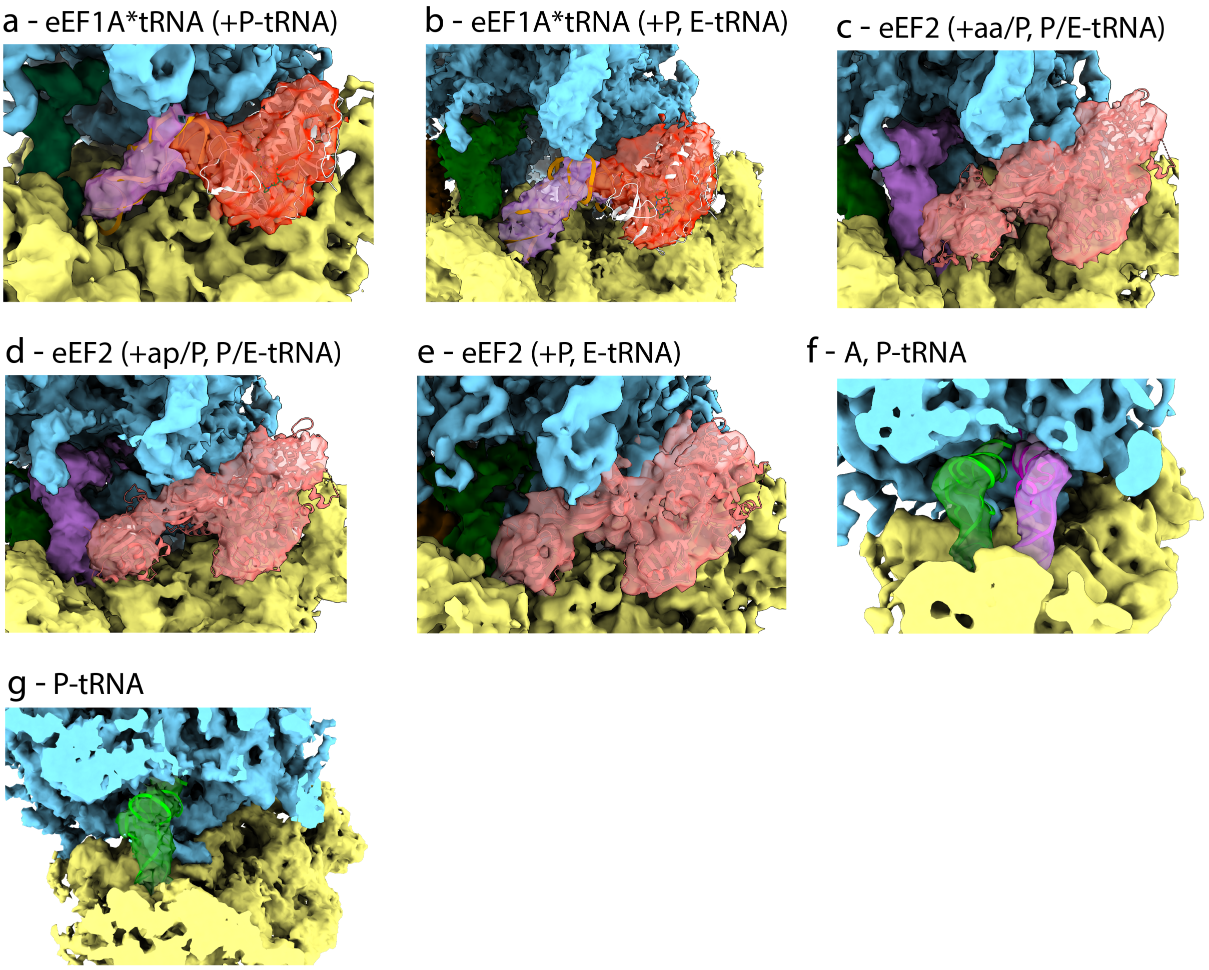

Supplementary Fig. 6. Fit of assigned elongation factors and tRNAs with corresponding ribosomal state maps shown in Fig. 3a.

(a, b) Atomic model of eEF1A*tRNA, PDB 5LZS [12], **(a)** rigid body fitted into the segmented density of 80S-eEF1A, A/T, P state map and **(b)** fitted into segmented density of 80S-eEF1A, A/T, P, E state map. eEF1A PDB model in white, A/T-tRNA PDB model in orange, A/T-tRNA cryo-ET density in lavender, eEF1A density in red and P-tRNA in green. **(c)**, **(d)** and **(e)** Atomic model of eEF2, PDB 7LS1 [13], rigid body fitted into the segmented density of **(c)** 80S-eEF2, aa/P, P/E state map**, (d)** 80S-eEF2, ap/P, P/E state map and **(e)** 80S-eEF2, P, E state. eEF2 PDB model shown in salmon, eEF2 map density in salmon, aa/P (and ap/P)-tRNA in lavender and P/E (and P)-tRNA in green. **(f)** Atomic models of A/A-tRNA and P/P-tRNA, PDB 6TNU [14], rigid body fitted into the segmented tRNA density of 80S-A,P state map. A/A-tRNA PDB model shown in pink, P/P-tRNA PDB model in light green, A/A-tRNA density in lavender, P/P-tRNA density in green. **(g)** Atomic model of P/P-tRNA, PDB 6TNU [14], rigid body fitted into the segmented tRNA density of 80S-P state map. P/P-tRNA PDB model is shown in light green and P/P-tRNA density in green. The 40S is shown in yellow, 60S in blue for all panels.

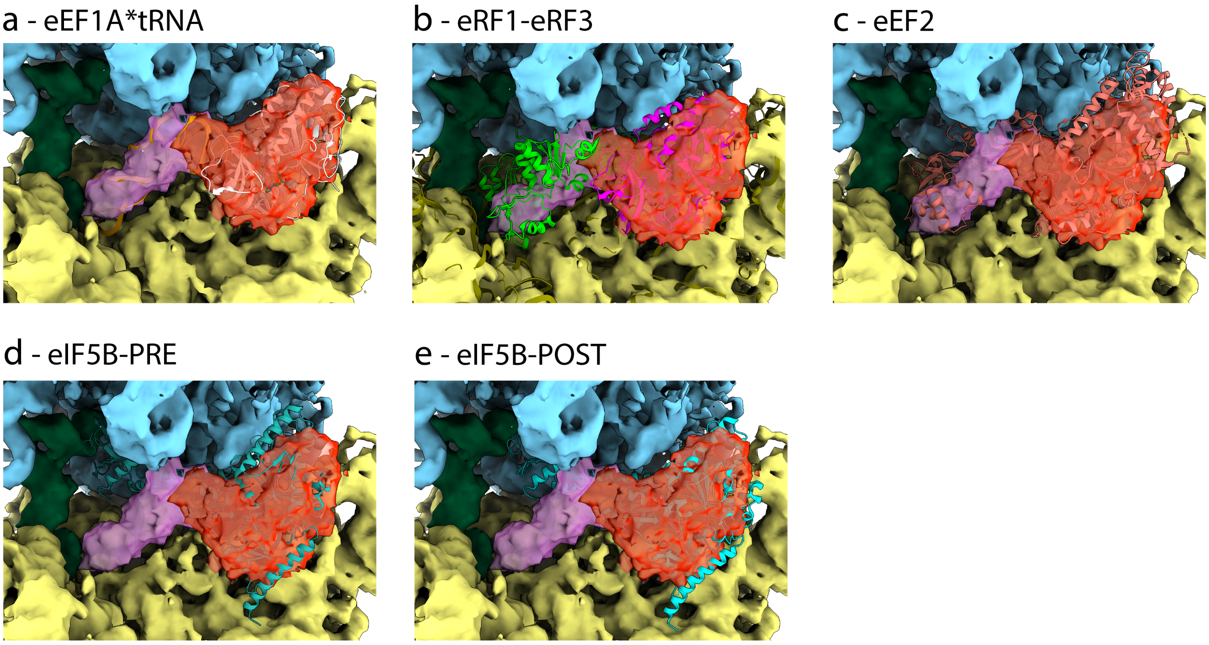

Supplementary Fig. 7. Comparison of fits with other small GTPase factors to the most prominent state map 80S-eEF1A, A/T, P.

(a) Assignment of 80S-eEF1A, A/T, P state based on rigid body fitting with atomic model of eEF1A*tRNA, PDB 5LZS [12]. eEF1A PDB model is shown in white, A/T-tRNA PDB model in orange (similar to Supplementary Fig. 6a). **(b), (c), (d), (e)** Rigid body fits of atomic models into segmented map densities as comparison to eEF1A fit in **(a)**. **(b)** Rigid body fit of eRF1-eRF3, PDB 4D61 [15]. eRF1 PDB model is shown in green, eRF3 PDB model in purple. **(c)** Rigid body fit of atomic model of eEF2, PDB 7LS1 [13]. eEF2 PDB model in salmon. **(d)** Rigid body fit of atomic model of eIF5B-PRE, PDB 4UJD [16]. eIF5B-PRE PDB model is shown in turquoise. **(e)** Rigid body fit of atomic model of eIF5B-POST, PDB 4UJC [16]. eIF5B-POST PDB model is shown in light blue. In all panels 40S is shown in yellow, 60S in blue, P-tRNA in green and map densities for fitting of small GTPase factors are shown lavender and red.

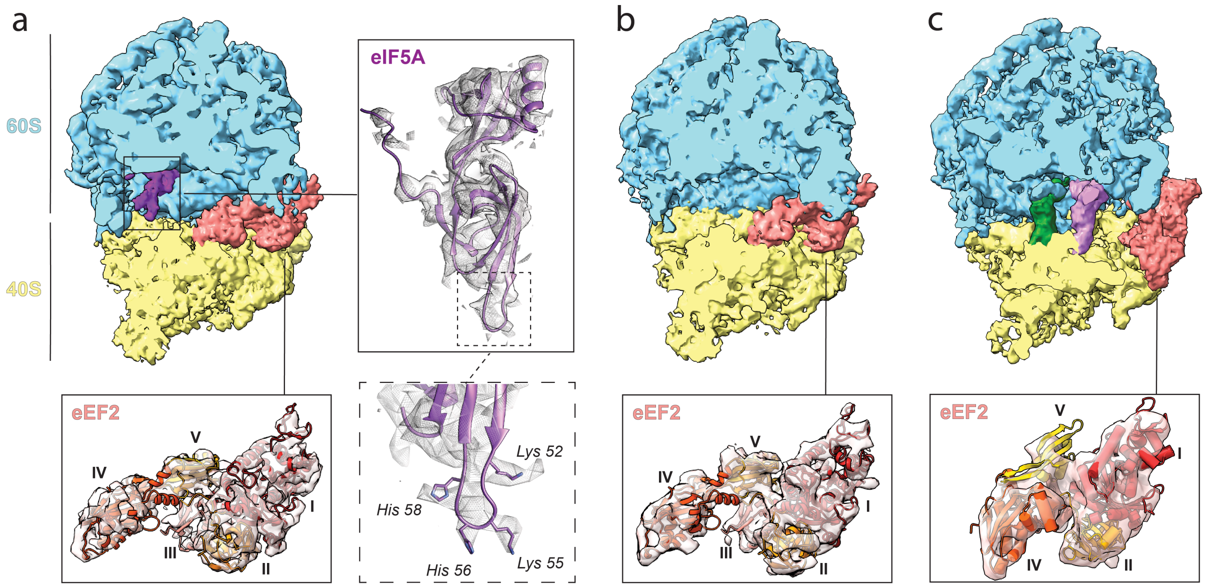

Supplementary Fig. 8. Putative factors of 80S ribosome states not assigned to the translation cycle. (a) Segmented volume of the 80S*eEF2 substate 1 structure showing the density at the eIF5A binding site (purple) and of eEF2 (salmon), and the fit of their Alphafold-predicted atomic models into the density. The models were rigid-body fitted and refined in ISOLDE. The original densities were low-pass filtered using b-factor 200. The density corresponding to eIF5A binding region is shown at lower threshold than the rest of the ribosome for representation reasons. To show the fit of eIF5A, original map and low-pass filtered maps are overlayed. **(b)** Segmented volume of the 80S*eEF2 substate 2 structure showing the density of eEF2 (salmon) and its fit, same as in **(a)**. **(c)** Individual domains of eEF2 (Alphafold-predicted atomic model) are rigid body fitted into the segmented density of 80S-factor-bound, A, P state map. eEF2 model domain 1 shown in red, domain 2 and 5 in yellow and domain 4 in orange, the cryo-ET density for the factor is shown in salmon, A-tRNA in lavender and P-tRNA in green. The domains of eEF2 are labeled in all insets.

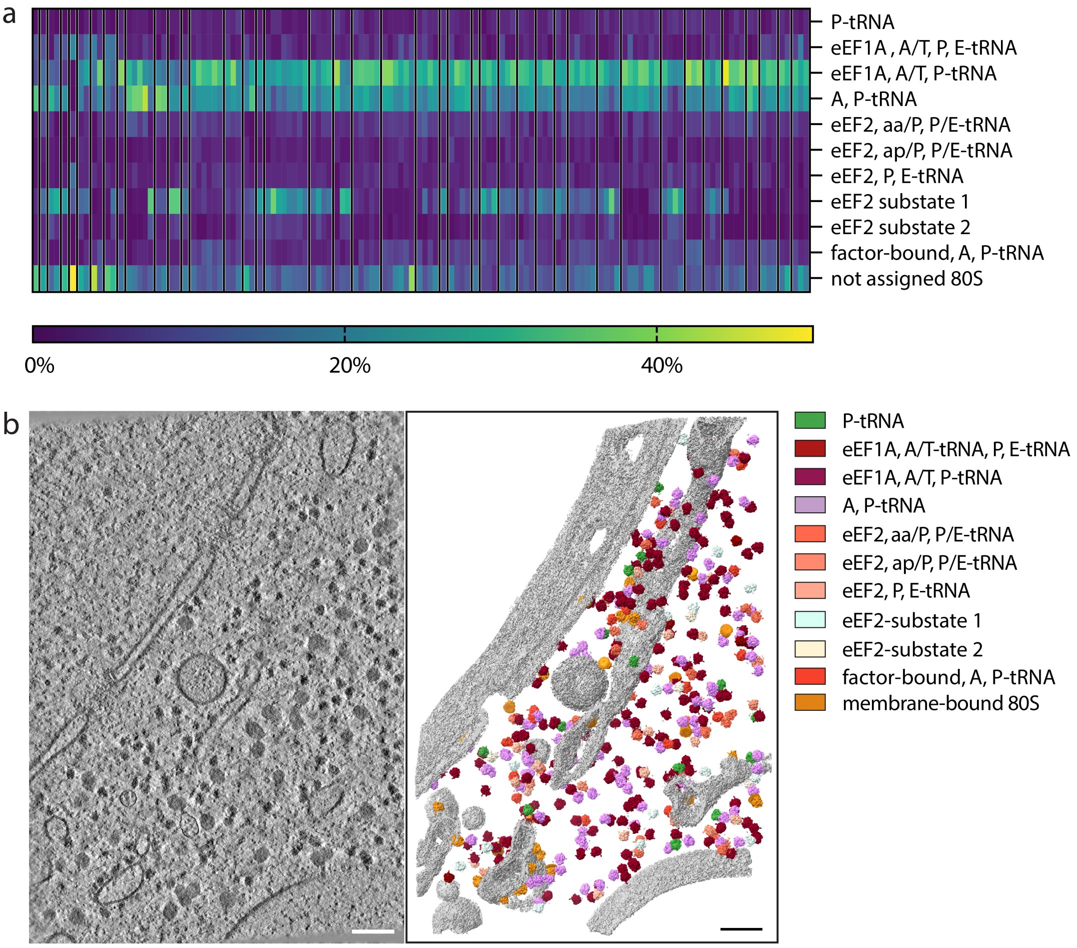

Supplementary Fig. 9. Distribution of ribosome classification across individual tomograms. (a) Heat map showing the percentage of individual ribosome translational states (see Fig. 3) and substates of 80S*eEF2 from the total of cytosolic ribosome particles. Each column represents one tomogram of dataset 1. Tomograms from different cells are additionally divided by black lines. Source data are provided as a Source Data file. **(b)** Spatial distribution of ribosome translational states in one representative tomogram out of 125 tomograms of dataset 1. Left panel: virtual slice of example tomogram (same as in Fig. 1d). Right panel: subtomogram averages of translational states placed into original position of the tomogram. Cellular membranes are shown as isosurface representation in grey. Scale bars: 100 nm.

Supplementary Table 1. Dataset parameters

	**Dataset 1**	**Dataset 2**
Microscope	Titan Krios G2 + FEG	Titan Krios G4 + cold FEG
Camera / EF	K3 / BioQuantum	Falcon4 / Selectris X
Nominal magnification	42000	105000
Pixel size (Å)	2.176	1.223
Defocus range (µm)	-2.5 to -5	-2.5 to -4.5
Dose rate (e/px/s)	10 - 20	3 - 6
Total dose (e/Å^2)	~132 - 150	~120
Number of datasets	5	1
Number of grids	14	1
Number of lamellae on grids	87	5
Number of cells in dataset	42	8
Number of TS	125	98
Number of particles used in final reconstruction	29858	24399
Nominal resolution (Å)	4.5	3.8
