# Supplementary material for: Structures of the eukaryotic ribosome and its translational states in situ
Source: Nat Commun. 2022 Dec 2;13:7435. doi: 10.1038/s41467-022-34997-w (PMC9718845; doi:10.1038/s41467-022-34997-w)
Supplement: Supplementary file 3 — Reporting Summary [file 41467_2022_34997_MOESM3_ESM.pdf]

nature portfolio

Corresponding author(s): Martin Beck

Last updated by author(s): Nov 11, 2022

Reporting Summary

Nature Portfolio wishes to improve the reproducibility of the work that we publish. This form provides structure for consistency and transparency in reporting. For further information on Nature Portfolio policies, see our Editorial Policies and the Editorial Policy Checklist.

Statistics

For all statistical analyses, confirm that the following items are present in the figure legend, table legend, main text, or Methods section.

n/a	Confirmed	
☐	☒	The exact sample size (*n*) for each experimental group/condition, given as a discrete number and unit of measurement
☐	☒	A statement on whether measurements were taken from distinct samples or whether the same sample was measured repeatedly
☒	☐	The statistical test(s) used AND whether they are one- or two-sided<br>*Only common tests should be described solely by name; describe more complex techniques in the Methods section.*
☒	☐	A description of all covariates tested
☒	☐	A description of any assumptions or corrections, such as tests of normality and adjustment for multiple comparisons
☐	☒	A full description of the statistical parameters including central tendency (e.g. means) or other basic estimates (e.g. regression coefficient) AND variation (e.g. standard deviation) or associated estimates of uncertainty (e.g. confidence intervals)
☒	☐	For null hypothesis testing, the test statistic (e.g. *F*, *t*, *r*) with confidence intervals, effect sizes, degrees of freedom and *P* value noted<br>*Give P values as exact values whenever suitable.*
☒	☐	For Bayesian analysis, information on the choice of priors and Markov chain Monte Carlo settings
☒	☐	For hierarchical and complex designs, identification of the appropriate level for tests and full reporting of outcomes
☒	☐	Estimates of effect sizes (e.g. Cohen's *d*, Pearson's *r*), indicating how they were calculated

Our web collection on statistics for biologists contains articles on many of the points above.

Software and code

Policy information about availability of computer code

Data collection	Software used for data collection was SerialEM (versions 3.8.1 and 4.0.1) (see methods part of manuscript).
Data analysis	Used software are described in the Methods section of the manuscript:<br>IMOD (versions 4.10.9 and 4.11.5), Relion (version 3.1), Warp, M (version 1.0.9), STOPGAP (https://github.com/williamnwan/STOPGAP/), dynamo2m (https://github.com/alisterburt/dynamo2m), ChimeraX, ModeRNA webservice, Coot, RNA Composer, forna package webservice, ISOLDE, subtomo2ChimeraX (https://github.com/builab/subtomo2Chimera), Amira-Avizo software (version 2021.1), GraphPad Prism 9, Matlab R2019b, Excel 2019

For manuscripts utilizing custom algorithms or software that are central to the research but not yet described in published literature, software must be made available to editors and reviewers. We strongly encourage code deposition in a community repository (e.g. GitHub). See the Nature Portfolio guidelines for submitting code & software for further information.

Data

Policy information about availability of data

All manuscripts must include a data availability statement. This statement should provide the following information, where applicable:

- Accession codes, unique identifiers, or web links for publicly available datasets
- A description of any restrictions on data availability
- For clinical datasets or third party data, please ensure that the statement adheres to our policy

Cryo-ET density maps generated in this study have been deposited in the EM Data Bank with the following accession codes: EMD-15807 [https://www.ebi.ac.uk/emdb/EMD-15807] (cytosolic-dataset2-high_res_map), EMD-15808 [https://www.ebi.ac.uk/emdb/EMD-15808] (cyotosolic-dataset1-high_res_map), EMD-15809 [https://www.ebi.ac.uk/emdb/EMD-15809] (membrane_bound), EMD-15810 [https://www.ebi.ac.uk/emdb/EMD-15810] (eEF1A, A/T, P), EMD-15811 [https://www.ebi.ac.uk/emdb/EMD-15811] (eEF1A, A/T, P, E), EMD-15812 [https://www.ebi.ac.uk/emdb/EMD-15812] (A, P), EMD-15813 [https://www.ebi.ac.uk/emdb/EMD-15813] (P), EMD-15814 [https://www.ebi.ac.uk/emdb/EMD-15814] (factor-bound, A, P), EMD-15815 [https://www.ebi.ac.uk/emdb/EMD-15815] (eEF2, aa/P, P/E), EMD-15816 [https://www.ebi.ac.uk/emdb/EMD-15816] (eEF2, ap/P, P/E), EMD-15843 [[https://www.ebi.ac.uk/emdb/EMD-15843]] (eEF2-substate1), EMD-15844 [https://www.ebi.ac.uk/emdb/EMD-15844] (eEF2-substate2) and EMD-15845 [https://www.ebi.ac.uk/emdb/EMD-15845] (eEF2, P, E).
The previously published structures EMD-4474 [https://www.ebi.ac.uk/emdb/EMD-4474] (S. cerevisiae 80S-Xrn), EMD-5591 [https://www.ebi.ac.uk/emdb/EMD-5591] (D. melanogaster 80S), EMD-5591 [https://www.ebi.ac.uk/emdb/EMD-5591] (M.musculus 80S-Ebp1), EMD-3069 [https://www.ebi.ac.uk/emdb/EMD-3069] (C. lupus 80S-Sec61-TRAP-OST), EMD-2644 [https://www.ebi.ac.uk/emdb/EMD-2644] (S. scrofa 80S-Sec61) (Supplementary Fig. 5g) , and EMD-0202 [https://www.ebi.ac.uk/emdb/EMD-0202] (S. cerevisiae 80S-NatA) are accessible through the Electron Microscopy Data Bank. The previously published ribosome structures 5LZS [http://doi.org/10.2210/pdb5LZS/pdb], 7LS1 [http://doi.org/10.2210/pdb7LS1/pdb], 6TNU [http://doi.org/10.2210/pdb6TNU/pdb], 4D61 [http://doi.org/10.2210/pdb4D61/pdb], 4UJC [http://doi.org/10.2210/pdb4UJC/pdb], 4UJD [http://doi.org/10.2210/pdb4UJC/pdb], 3J7R [http://doi.org/10.2210/pdb3j7r/pdb], 4V6W [http://doi.org/10.2210/pdb4v6w/pdb], 5ANB [http://dx.doi.org/10.2210/pdb5anb/pdb] and 7OYC [http://doi.org/10.2210/pdb7OYC/pdb] are available through the Protein Data Base. The templates for ribosomal RNA secondary structure diagrams were downloaded from CRW database [https://crw-site.chemistry.gatech.edu/]. Source data are provided with this paper.

Human research participants

Policy information about studies involving human research participants and Sex and Gender in Research.

Reporting on sex and gender	Study does not involve human research participants.
Population characteristics	Study does not involve human research participants.
Recruitment	Study does not involve human research participants.
Ethics oversight	Study does not involve human research participants.

Note that full information on the approval of the study protocol must also be provided in the manuscript.

Field-specific reporting

Please select the one below that is the best fit for your research. If you are not sure, read the appropriate sections before making your selection.

☒ Life sciences ☐ Behavioural & social sciences ☐ Ecological, evolutionary & environmental sciences

For a reference copy of the document with all sections, see nature.com/documents/nr-reporting-summary-flat.pdf

Life sciences study design

All studies must disclose on these points even when the disclosure is negative.

Sample size	We did not pre-determine the sample size when the study was being designed. We acquired the maximum amount of cryo-ET data from the available samples within the available microscope time.
Data exclusions	A fraction of cryo-ET data was excluded due to acquisition problems, or due to low quality leading to problems in alignment and tomogram reconstruction.
Replication	Two separate cryo-ET datasets were acquired and processed independently of each other to determine the ribosome structure. Cryo-ET samples were prepared from separately pre-cultured cells, which were cryo-fixed in 10 independent plunge freezing sessions (9 for dataset 1, 1 for dataset 2). Dataset 1 was acquired in 5 separate 48 hr microscope session on 14 EM grids with in total 87 lamellae. We selected 125 well-aligned tomograms from 42 cells for subtomogram averaging. Dataset 2 was acquired in one 48 hr microscope session from 1 EM grid

with 5 lamellae. We selected a total of 98 tomograms from 8 cells for subtomogram averaging. All details are additionally mentioned in the Methods section and Supplementary table 1.

Randomization	We did not use randomization in our study.
Blinding	We did not use blinding in our study..

Reporting for specific materials, systems and methods

We require information from authors about some types of materials, experimental systems and methods used in many studies. Here, indicate whether each material, system or method listed is relevant to your study. If you are not sure if a list item applies to your research, read the appropriate section before selecting a response.

Materials & experimental systems

n/a	Involved in the study
☒	☐ Antibodies
☐	☒ Eukaryotic cell lines
☒	☐ Palaeontology and archaeology
☒	☐ Animals and other organisms
☒	☐ Clinical data
☒	☐ Dual use research of concern

Methods

n/a	Involved in the study
☒	☐ ChIP-seq
☒	☐ Flow cytometry
☒	☐ MRI-based neuroimaging

Eukaryotic cell lines

Policy information about cell lines and Sex and Gender in Research

Cell line source(s)	The D.discoideum cell line (GFP-Nup62) used in this study was derived from the Ax2-214 strain of the Gerisch Lab (MPI Martinsried, Germany). The Ax2-214 strain is available through dictybase.
Authentication	The D.discoideum cell line used in this study was not authenticated.
Mycoplasma contamination	Cell lines were not tested for mycoplasma contamination, since it is not relevant to D.discoideum.
Commonly misidentified lines (See ICLAC register)	None
